# Atezolizumab plus Bevacizumab with or without Transarterial Chemoembolization as First-Line Treatment for Barcelona Clinic Liver Cancer Stage C Hepatocellular Carcinoma: A Nationwide Multicenter Retrospective Study

**DOI:** 10.1159/lic/admag010

**Published:** 2026-09-05

**Authors:** Xiao-Wu Zhang, Li-Wei Deng, Yin-Gen Luo, Yin-Fa Xie, Rui-Bao Liu, Hai-Bo Shao, Tong-Guo Si, Dui-Ping Feng, Jian Wang, Yu Zhang, Qing-Dong Li, Hong-Tao Hu, Wei-Jun Fan, Peng Song, Li-Nan Yin, Yan Wang, Yi Chen, Tian-Shi Lv, Yong Xie, Tian-Yi Hua, Jing Xiao, Rui-Can Nie, Lu-Jun Shen, Zhi-Ming Xuan, Chang Liu, Xue-Gang Yang, Xu Chang, Xiao Li

**Affiliations:** a Department of Interventional Therapy, National Cancer Center/National Clinical Research Center for Cancer/Cancer Hospital, Chinese Academy of Medical Sciences and Peking Union Medical College, Beijing, China; b Department of Interventional Therapy II, Shandong Cancer Hospital and Institute, Shandong First Medical University and Shandong Academy of Medical Sciences, Jinan, China; c Department of Interventional Radiology, Harbin Medical University Cancer Hospital, Harbin, China; d Department of Interventional Radiology, The First Hospital of China Medical University, Liaoning, China; e Department of Interventional Treatment, Tianjin Cancer Hospital Airport Hospital, National Clinical Research Center for Cancer, Tianjin’s Clinical Research Center for Cancer , Tianjin, China; f Department of Interventional Therapy, Tianjin Medical University Cancer Institute and Hospital, National Clinical Research Center for Cancer, Key Laboratory of Cancer Prevention and Therapy, Tianjin’s Clinical Research Center for Cancer, Tianjin, China; g Department of Oncology and Vascular Intervention, First Hospital of Shanxi Medical University, Shanxi Provincial Clinical Research Center for Interventional Medicine, Taiyuan, China; h Department of Interventional Radiology and Vascular Surgery, Peking University First Hospital, Beijing, China; i Department of Hepatobiliary and Pancreatic Surgery, Sichuan Provincial People's Hospital, University of Electronic Science and Technology of China, Chengdu, China; j Vascular and Interventional Department, Chongqing University Cancer Hospital, Chongqing, China; k Department of Interventional Radiology, The Affiliated Cancer Hospital of Zhengzhou University, Henan Cancer Hospital, Zhengzhou, China; l Department of Minimally Invasive Interventional Therapy, Sun Yat-sen University Cancer Center, Guangzhou, China; m Department of Interventional Therapy, National Cancer Center/National Clinical Research Center for Cancer/Cancer Hospital and Shenzhen Hospital, Chinese Academy of Medical Sciences and Peking Union Medical College, Shenzhen, China; n Intervention Center, West China Hospital, Sichuan University, Chengdu, China; o Department of Interventional Therapy, Sichuan Clinical Research Center for Cancer, Sichuan Cancer Hospital & Institute, Sichuan Cancer Center, University of Electronic Science and Technology of China, Chengdu, China

**Keywords:** Hepatocellular carcinoma, Transarterial chemoembolization, Atezolizumab, Bevacizumab, Combined modality therapy

## Abstract

**Introduction:**

Atezolizumab plus bevacizumab (Atezo/Bev) has been recommended globally for patients with Barcelona Clinic Liver Cancer (BCLC) stage C hepatocellular carcinoma (HCC); however, this regimen has shown modest survival benefits. Therefore, this study aimed to assess the efficacy and safety of Atezo/Bev with or without transarterial chemoembolization (TACE) as first-line treatment for BCLC C HCC.

**Methods:**

This nationwide multicenter retrospective cohort study recruited patients with BCLC stage C HCC from 14 medical centres who received TACE combined with Atezo/Bev (TACE+Atezo/Bev) or Atezo/Bev alone as first-line treatment between October 2020 and January 2025. To mitigate potential confounding imbalances between groups, propensity score matching (PSM) and stabilized inverse probability of treatment weighting (sIPTW) were applied. Overall survival (OS) was set as the primary endpoint, with progression-free survival (PFS), objective response rate (ORR), and safety assessed as the secondary endpoints.

**Results:**

Of the 256 patients included in the analysis, 154 received TACE+Atezo/Bev and 102 received Atezo/Bev. After PSM, analysis of 96 patients per group showed that the TACE+Atezo/Bev group significantly extended the median OS (25.9 months vs. 15.2 months, *p* < 0.001) and PFS (15.8 months vs. 7.4 months, *p* < 0.001), compared with the Atezo/Bev group. Post-application of sIPTW, 154 patients were assigned to the TACE+Atezo/Bev group and 102 to the Atezo/Bev group, and the median OS (25.3 vs. 15.4 months, *p* < 0.001) and PFS (15.5 vs. 7.5 months, *p* < 0.001) were significantly longer in the TACE+Atezo/Bev group than in the Atezo/Bev group. A higher ORR was observed in the TACE+Atezo/Bev group compared with the Atezo/Bev group (primary cohort: 48.7% vs. 31.4%, *p* = 0.009; PSM cohort: 51.0% vs. 31.2%, *p* = 0.008; sIPTW cohort: 50.6% vs. 32.0%, *p* = 0.005). Moreover, Grade 3/4 adverse events (AEs) occurred in 60 patients (39.0%) in the TACE+Atezo/Bev group and 34 patients (33.3%) in the Atezo/Bev group.

**Conclusion:**

Compared with Atezo/Bev alone, TACE+Atezo/Bev may significantly improve ORR, PFS, and OS as first-line treatment for BCLC stage C HCC, while maintaining an acceptable safety profile.

## Introduction

Liver cancer is the sixth most prevalent cancer and the third leading cause of cancer-associated deaths worldwide, while China accounts for nearly half of the global liver cancer burden [1, 2]. Hepatocellular carcinoma (HCC) dominates as the primary liver cancer type, comprising 85–90% of cases [3]. More than 50% of patients with HCC are diagnosed at Barcelona Clinic Liver Cancer (BCLC) stage C due to its insidious onset and lack of effective early diagnostic markers, consequently missing the opportunity for curative treatment and resulting in a poor prognosis [4–6].

In recent years, the combination of immunotherapies and antiangiogenic therapies has revolutionized the treatment landscape of advanced HCC [7, 8]. Particularly, the success of the Phase III clinical trial (IMbrave150 trial) demonstrated the superiority of the combination of atezolizumab and bevacizumab (Atezo/Bev) over sorafenib in the first-line treatment of unresectable HCC, thus leading to its approval as the standard first-line treatment worldwide [9]. However, in the updated analysis of the IMbrave150 trial, the efficacy of Atezo/Bev was unsatisfactory in the subgroup of patients with BCLC stage C HCC, with median progression-free survival (PFS) of 6.5 months and objective response rate (ORR) of 27% [10]. Therefore, there is an urgent necessity to explore other effective therapeutic strategies in combination with atezolizumab and bevacizumab to improve the prognosis of patients with BCLC stage C HCC.

In the China Liver Cancer (CNLC) staging system, HCC at BCLC stage C is further stratified into stage IIIa, defined by vascular invasion without extrahepatic metastasis, and stage IIIb, defined by extrahepatic metastasis [3]. CNLC stage IIIa is eligible for transarterial chemoembolization (TACE), and TACE may be considered for CNLC stage IIIb when control of intrahepatic tumour growth is expected to provide clinical benefit; these recommendations are supported by several guidelines and expert consensus [11–13]. However, TACE may promote tumour angiogenesis by upregulating vascular endothelial growth factor (VEGF) receptor to induce tumour metastasis and recurrence [14]. As an antiangiogenic agent, bevacizumab can inhibit the activation of the VEGF signalling pathway and reduce the formation of tumour neovascularization [15]. A prior phase II clinical trial demonstrated that concurrent administration of TACE plus bevacizumab exhibited antitumour activity in patients with unresectable HCC [16]. In addition, TACE may stimulate immune responses by inducing tumour necrosis and releasing tumour-specific antigens to improve the therapeutic efficacy of immunotherapy [17–19]. So in theory, the combination of Atezo/Bev and TACE has a promising synergistic effect for BCLC stage C HCC. This nationwide multicenter retrospective cohort study evaluated the efficacy and safety of TACE combined with Atezo/Bev (TACE+Atezo/Bev) as first-line treatment for patients with BCLC stage C HCC.

## Methods

### Patients

This nationwide multicenter retrospective cohort study enrolled patients with BCLC stage C HCC treated with Atezo/Bev with or without TACE as first-line treatment from October 2020 to January 2025 at 14 hospitals in China. The inclusion criteria were as follows: (1) age 18–80 years; (2) the diagnosis of HCC was confirmed according to the histological evidence or the American Association for the Study of Liver Diseases (AASLD) guideline [20]; (3) Eastern Cooperative Oncology Group performance status (ECOG PS) < 2; (4) Child-Pugh class A or B; (5) possession of at least 1 measurable intrahepatic lesion as per modified Response Evaluation Criteria in Solid Tumours (mRECIST); and (6) for patients in the TACE+Atezo/Bev group, the initial TACE session was performed within 1 month before or after initiation of atezolizumab plus bevacizumab, with at least one cycle of atezolizumab plus bevacizumab administered after the initial TACE session. The exclusion criteria included the following: (1) BCLC stage A and B; (2) prior history of any antitumour treatment (systemic or locoregional therapy); (3) concurrent other malignancies within 5 years; (4) presence of uncontrollable coagulation disorders or refractory ascites; (5) significant oesophageal varices or observable red wale marks; and (6) loss to follow-up or incomplete medical records.

We gathered each patient’s baseline details, including age, sex, ECOG PS, aetiology, cirrhosis, tumour size, tumour number, portal vein tumoural thrombosis (PVTT), extrahepatic spread (EHS), albumin-bilirubin (ALBI) grade, Child-Pugh class, alpha-fetoprotein (AFP), portal hypertension (PHT), and ascites.

### TACE Procedure

Eligibility for TACE was determined at treatment initiation, and all TACE procedures were performed in accordance with Chinese guidelines and standardized protocols implemented across the participating hospitals. In a subset of patients, Atezo/Bev was initiated before the planned TACE procedure, which was subsequently implemented through interdepartmental coordination. For patients with Vp3/4 PVTT, TACE was performed only after confirming acceptable performance status, preserved hepatic reserve, adequate portal venous flow or collateral compensation, and identifiable tumour feeding arteries. For patients with EHS, TACE was considered when intrahepatic disease represented the dominant tumour burden and clinical benefit was expected from controlling intrahepatic tumour growth [3, 21]. All tumour-feeding arteries, encompassing their origins and any ectopic or collateral blood supplies, were fully visualized, and all TACE procedures were performed via superselective catheterization and embolization. The selection and dosage of embolic and chemotherapeutic agents were determined jointly by physicians and patients in accordance with guidelines [21]. The choice of conventional TACE (cTACE) or drug-eluting beads TACE (DEB-TACE) was determined at each participating centre by the treating interventional radiologist or local multidisciplinary team on the basis of guideline recommendations together with patient related considerations, including clinical condition, treatment preference and financial considerations [3, 21].

For cTACE, Lipiodol (2–20 mL) was mixed with chemotherapeutic drugs to form a stable “water-in-oil” emulsion, which was subsequently infused into the tumour-supplying arteries. When needed, particulate embolic agents (such as blank microspheres, gelatin sponge particles, or polyvinyl alcohol particles) were used for further embolization to attain a satisfactory endpoint. For DEB-TACE, Callispheres Beads (Jiangsu Hengrui Medicine Co., Ltd., Jiangsu, China) or HepaSpheres Beads (Biosphere Medical, Inc., South Jordan, UT) with a diameter of 100–300 or 300–500 μm loaded with chemotherapeutic drugs, were mixed with non-ionic contrast agents at a 1:1 ratio and subsequently infused into the tumour-feeding arteries. Injection was completed upon achievement of contrast agent stasis.

Subsequently, if the presence of viable tumour or intrahepatic de novo lesions was detected by enhanced computed tomography (CT) or magnetic resonance imaging (MRI), TACE could be repeated. Cessation of TACE was determined by the satisfaction of any of the following criteria at every participating centre: (1) severe hepatic deterioration with a Child-Pugh score exceeding 9, uncontrollable ascites, coagulation disorders or overt hepatic encephalopathy; (2) ECOG PS >2; or (3) continuous progression following three sessions of TACE.

### Atezolizumab/Bevacizumab

Intravenous administration of 1,200 mg atezolizumab and 15 mg/kg bevacizumab was conducted every 3 weeks. According to the guidance of the drug instructions, the treatment regimen was dose-reduced, interrupted or replaced upon the occurrence of intolerable treatment-related adverse events (TRAEs) or disease progression.

### Follow-Up and Assessments

A series of follow-up evaluations, including clinical symptoms, liver contrast-enhanced CT or MRI, lung CT scan, laboratory testing, and assessment of TRAEs, were conducted at 4–8 weeks intervals. The final follow-up occurred on July 30, 2025.

According to the mRECIST, treatment responses were subsequently assessed by two independent radiologists with at least 10 years of experience at each participating centre. The primary endpoint was overall survival (OS), defined as the interval from initiation of atezolizumab plus bevacizumab to death from any cause or last follow-up. The secondary endpoints included PFS, ORR and TRAEs. PFS was defined as the interval from initiation of atezolizumab plus bevacizumab to first radiological evidence of tumour progression or death from any cause. ORR was considered the sum of complete response (CR) and partial response (PR). The Common Terminology Criteria for Adverse Events (version 5.0) was applied to evaluate TRAEs. PHT was clinically defined as the presence of oesophagogastric varices on endoscopy or baseline imaging, or splenomegaly accompanied by a platelet count <100 × 10^9^/L.

### Statistical Analysis

Statistical analyses were performed using R software (version 4.4.1), with a two-tailed *p* value <0.05 considered statistically significant. The standardized mean difference (SMD) was employed to evaluate potential baseline imbalances between groups as it eliminates the influence of measurement units and sample sizes [22]. An SMD less than 0.1 was defined as indicating a negligible difference. One to one propensity score matching (PSM) without replacement was performed using a caliper of 0.2, with propensity scores estimated from a multivariable logistic regression model including baseline covariates: sex, age, ECOG PS, aetiology, tumour number, tumour size, PVTT, EHS, cirrhosis, AFP, and Child-Pugh class. The application of stabilized inverse probability of treatment weighting (sIPTW), calculated from propensity scores, effectively mitigated potential baseline imbalances (SMDs of all baseline variables were less than 0.1). Factors identified as statistically significant (*p* < 0.05) through univariate analysis were incorporated into the multivariate Cox proportional-hazards regression model for identifying independent factors. PFS and OS were estimated using Kaplan-Meier curves. To account for time dependent TACE exposure, an sIPTW weighted time dependent Cox model and a landmark sIPTW analysis at 1 month were performed as OS sensitivity analyses. Among patients in the TACE+Atezo/Bev group, exploratory comparisons between cTACE and DEB-TACE were assessed using multivariable Cox regression adjusted for the same prespecified baseline covariates.

## Results

### Patient Characteristics

The primary cohort comprised a total of 256 patients, with 154 in the TACE+Atezo/Bev group and 102 in the Atezo/Bev group (Fig. 1). Following PSM, 96 patients in each group were analysed. After applying sIPTW, 154 patients were assigned to the TACE+Atezo/Bev group and 102 to the Atezo/Bev group (Table 1). The clinical characteristics of the two groups were balanced through PSM and sIPTW, as assessed by the SMD for all baseline variables before and after adjustment and the propensity score distribution after weighting (online suppl. Figs. S1 and S2). Treatments administered following first-line treatment and subsequent treatments after progression are summarized in online supplementary Table 1. The TACE+Atezo/Bev group comprised 90 patients treated with cTACE and 64 treated with DEB-TACE, with baseline characteristics according to TACE type summarized in online supplementary Table 2.

**Fig. 1.**
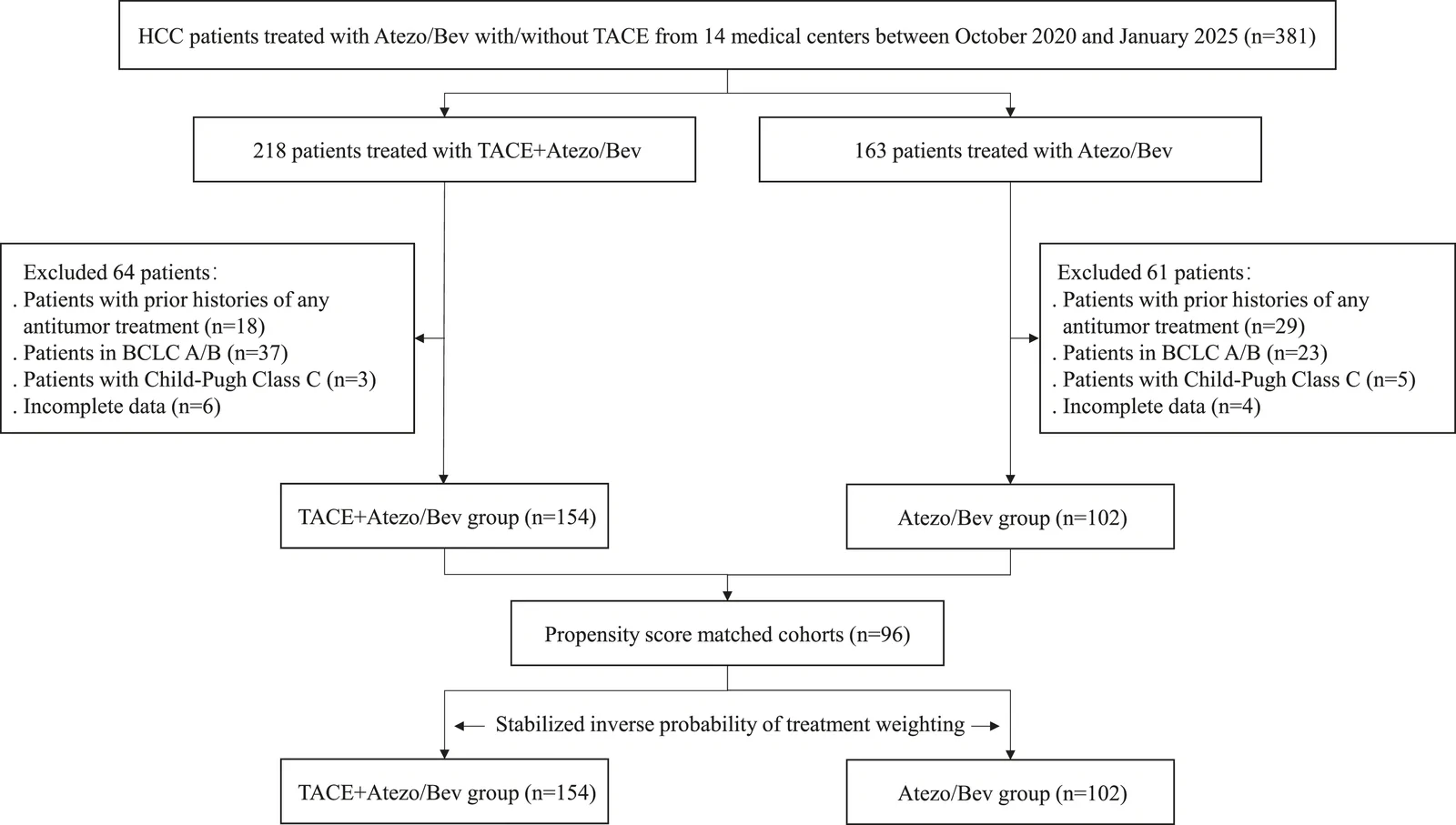
Flowchart of the study. HCC, hepatocellular carcinoma; Atezo/Bev, atezolizumab and bevacizumab; TACE, transarterial chemoembolization; BCLC, Barcelona Clinic Liver Cancer.

**Table 1.** Baseline characteristics of patients in the two groups

​	Primary cohort				PSM cohort				sIPTW cohort			
	TACE+Atezo/Bev (N = 154)	Atezo/Bev (N = 102)	*p* value	SMD	TACE+Atezo/Bev (N = 96)	Atezo/Bev (N = 96)	*p* value	SMD	TACE+Atezo/Bev (N = 154)	Atezo/Bev (N = 102)	*p* value	SMD
Age, years	​	​	1.000	0.002	​	​	0.999	0.022	​	​	0.932	0.011
<60	98 (63.6)	65 (63.7)	​	​	61 (63.5)	62 (64.6)	​	​	98 (63.8)	66 (64.3)	​	​
≥60	56 (36.4)	37 (36.3)	​	​	35 (36.5)	34 (35.4)	​	​	56 (36.2)	36 (35.7)	​	​
Sex	​	​	0.433	0.124	​	​	0.999	0.032	​	​	0.930	0.011
Male	139 (90.3)	88 (86.3)	​	​	85 (88.5)	84 (87.5)	​	​	136 (88.6)	90 (88.3)	​	​
Female	15 (9.7)	14 (13.7)	​	​	11 (11.5)	12 (12.5)	​	​	18 (11.4)	12 (11.7)	​	​
ECOG PS	​	​	0.524	0.098	​	​	0.999	0.021	​	​	0.881	0.020
0	89 (57.8)	54 (52.9)	​	​	50 (52.1)	51 (53.1)	​	​	87 (56.6)	59 (57.6)	​	​
1	65 (42.2)	48 (47.1)	​	​	46 (47.9)	45 (46.9)	​	​	67 (43.4)	43 (42.4)	​	​
Aetiology	​	​	0.463	0.111	​	​	0.752	0.068	​	​	0.964	0.006
Others	42 (27.3)	33 (32.4)	​	​	27 (28.1)	30 (31.2)	​	​	45 (29.1)	30 (29.4)	​	​
HBV	112 (72.7)	69 (67.6)	​	​	69 (71.9)	66 (68.8)	​	​	109 (70.9)	72 (70.6)	​	​
Cirrhosis	​	​	0.123	0.219	​	​	0.617	0.096	​	​	0.847	0.026
Absent	48 (31.2)	22 (21.6)	​	​	26 (27.1)	22 (22.9)	​	​	42 (27.0)	27 (25.9)	​	​
Present	106 (68.8)	80 (78.4)	​	​	70 (72.9)	74 (77.1)	​	​	112 (73.0)	75 (74.1)	​	​
Tumour size, cm	​	​	0.048	0.269	​	​	0.630	0.093	​	​	0.927	0.012
<5	33 (21.4)	34 (33.3)	​	​	25 (26.0)	29 (30.2)	​	​	41 (26.4)	28 (27.0)	​	​
≥5	121 (78.6)	68 (66.7)	​	​	71 (74.0)	67 (69.8)	​	​	113 (73.6)	74 (73.0)	​	​
Tumour number	​	​	0.396	0.126	​	​	0.999	<0.001	​	​	0.860	0.024
≤3	41 (26.6)	33 (32.4)	​	​	30 (31.2)	30 (31.2)	​	​	45 (29.4)	31 (30.4)	​	​
>3	113 (73.4)	69 (67.6)	​	​	66 (68.8)	66 (68.8)	​	​	109 (70.6)	71 (69.6)	​	​
PVTT	​	​	0.368	0.132	​	​	0.999	<0.001	​	​	0.996	<0.001
Absent	42 (27.3)	34 (33.3)	​	​	32 (33.3)	32 (33.3)	​	​	46 (30.0)	31 (30.0)	​	​
Present	112 (72.7)	68 (66.7)	​	​	64 (66.7)	64 (66.7)	​	​	108 (70.0)	71 (70.0)	​	​
Vp1	6 (3.9)	3 (2.9)	​	​	4 (4.2)	3 (3.1)	​	​	6 (3.8)	4 (3.9)	​	​
Vp2	22 (14.3)	11 (10.8)	​	​	10 (10.4)	11 (11.5)	​	​	20 (13.3)	13 (13.1)	​	​
Vp3	57 (37.0)	35 (34.3)	​	​	35 (36.5)	32 (33.3)	​	​	55 (35.4)	35 (34.3)	​	​
Vp4	27 (17.5)	19 (18.6)	​	​	15 (15.6)	18 (18.8)	​	​	27 (17.4)	19 (18.6)	​	​
EHS	​	​	0.128	0.212	​	​	0.662	0.084	​	​	0.982	0.003
Absent	75 (48.7)	39 (38.2)	​	​	43 (44.8)	39 (40.6)	​	​	70 (45.7)	46 (45.5)	​	​
Present	79 (51.3)	63 (61.8)	​	​	53 (55.2)	57 (59.4)	​	​	84 (54.3)	56 (54.5)	​	​
Child-Pugh class	​	​	0.453	0.115	​	​	0.999	0.025	​	​	0.962	0.006
A	125 (81.2)	78 (76.5)	​	​	73 (76.0)	74 (77.1)	​	​	122 (79.5)	81 (79.2)	​	​
B	29 (18.8)	24 (23.5)	​	​	23 (24.0)	22 (22.9)	​	​	32 (20.5)	21 (20.8)	​	​
ALBI grade	​	​	0.895	0.033	​	​	0.999	0.021	​	​	0.957	0.007
1	72 (46.8)	46 (45.1)	​	​	41 (42.7)	42 (43.8)	​	​	71 (46.1)	47 (46.5)	​	​
2/3	82 (53.2)	56 (54.9)	​	​	55 (57.3)	54 (56.2)	​	​	83 (53.9)	55 (53.5)	​	​
AFP, ng/mL	​	​	0.558	0.091	​	​	0.885	0.042	​	​	0.968	0.005
<400	84 (54.5)	51 (50.0)	​	​	52 (54.2)	50 (52.1)	​	​	81 (52.6)	54 (52.9)	​	​
≥400	70 (45.5)	51 (50.0)	​	​	44 (45.8)	46 (47.9)	​	​	73 (47.4)	48 (47.1)	​	​
PHT	​	​	0.344	0.137	​	​	0.885	0.042	​	​	0.626	0.064
Absent	80 (51.9)	46 (45.1)	​	​	46 (47.9)	44 (45.8)	​	​	75 (48.6)	46 (45.4)	​	​
Present	74 (48.1)	56 (54.9)	​	​	50 (52.1)	52 (54.2)	​	​	79 (51.4)	56 (54.6)	​	​
Ascites	​	​	1.000	0.017	​	​	0.757	0.067	​	​	0.495	0.089
Absent	106 (68.8)	71 (69.6)	​	​	64 (66.7)	67 (69.8)	​	​	104 (67.5)	73 (71.6)	​	​
Present	48 (31.2)	31 (30.4)	​	​	32 (33.3)	29 (30.2)	​	​	50 (32.5)	29 (28.4)	​	​

PSM, propensity score matching; sIPTW, stabilized inverse probability of treatment weighting; TACE, transarterial chemoembolization; Atezo/Bev, atezolizumab plus bevacizumab; SMD, standardized mean difference; ECOG PS, Eastern Cooperative Oncology Group performance status; HBV, hepatitis B virus; PVTT, portal vein tumoral thrombosis; EHS, extrahepatic spread; ALBI, albumin-Bilirubin; AFP, alpha-fetoprotein; PHT, portal hypertension.

### Efficacy

The median follow-up was 26.9 months (95% confidence interval [CI], 25.3–33.7) in the TACE+Atezo/Bev group and 35.3 months (95% CI, 33.5-not reached [NR]) in the Atezo/Bev group, with no statistically significant difference between the two groups (*p* = 0.543). During the follow-up, 72 of 154 patients (46.8%) in the TACE+Atezo/Bev group and 72 of 102 patients (70.6%) in the Atezo/Bev group died. Compared with the Atezo/Bev group, the TACE+Atezo/Bev group exhibited a higher ORR in the primary (48.7% vs. 31.4%, *p* = 0.009), PSM (51.0% vs. 31.2%, *p* = 0.008) and sIPTW (50.6% vs. 32.0%, *p* = 0.005) cohort respectively (online suppl. Table S3).

In the primary cohort, the median PFS and OS were significantly improved in the TACE+Atezo/Bev group versus the Atezo/Bev group (median PFS: 14.7 months [95% CI: 13.0–22.4] vs. 7.5 months [95% CI: 6.8–11.1]; *p* < 0.001; hazard ratios [HR]: 0.50, 95% CI: 0.37–0.68; median OS: 24.3 months [95% CI: 21.7–45.7] vs. 15.2 months [95% CI: 14.2–20.2]; *p* < 0.001; HR: 0.46, 95% CI: 0.33–0.65) (online suppl. Fig. S3).

After PSM, the median PFS and OS of the TACE+Atezo/Bev group were significantly longer than those of the Atezo/Bev group (median PFS: 15.8 months [95% CI: 13.9–50.4] vs. 7.4 months [95% CI: 6.4–11.1]; *p* < 0.001; HR: 0.47, 95% CI: 0.33–0.67; median OS: 25.9 months [95% CI: 21.1–NR] vs. 15.2 months [95% CI: 14.2–17.5]; *p* < 0.001; HR: 0.37, 95% CI: 0.25–0.55) (Fig. 2).

**Fig. 2.**
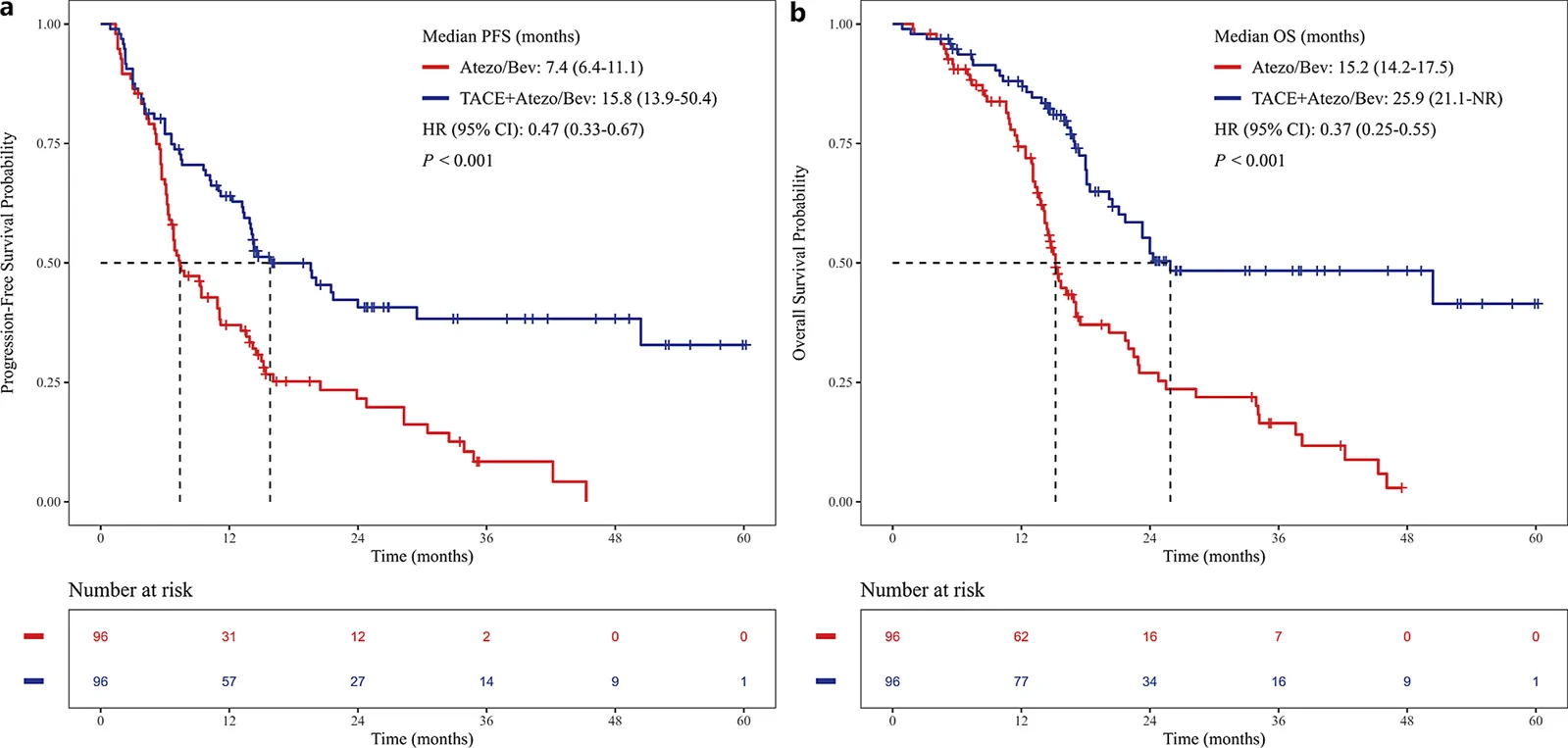
Kaplan-Meier curves of progression-free survival (**a**) and overall survival (**b**) in the two groups after propensity score matching. Atezo/Bev, atezolizumab and bevacizumab; TACE, transarterial chemoembolization.

Following sIPTW, the median PFS and OS were also longer in the TACE+Atezo/Bev group than in the Atezo/Bev group (median PFS: 15.5 months [95% CI: 13.3–24.0] vs. 7.5 months [95% CI: 6.6–11.1]; *p* < 0.001; HR: 0.49, 95% CI: 0.36–0.67; median OS: 25.3 months [95% CI: 22.4–50.4] vs. 15.4 months [95% CI: 14.2–20.2]; *p* < 0.001; HR: 0.44, 95% CI: 0.31–0.63) (Fig. 3). Sensitivity analyses addressing time dependent TACE exposure, including an sIPTW weighted time dependent Cox model and a landmark sIPTW analysis at 1 month, further supported the OS benefit of TACE plus Atezo/Bev over Atezo/Bev alone (online suppl. Figs. S4 and S5).

**Fig. 3.**
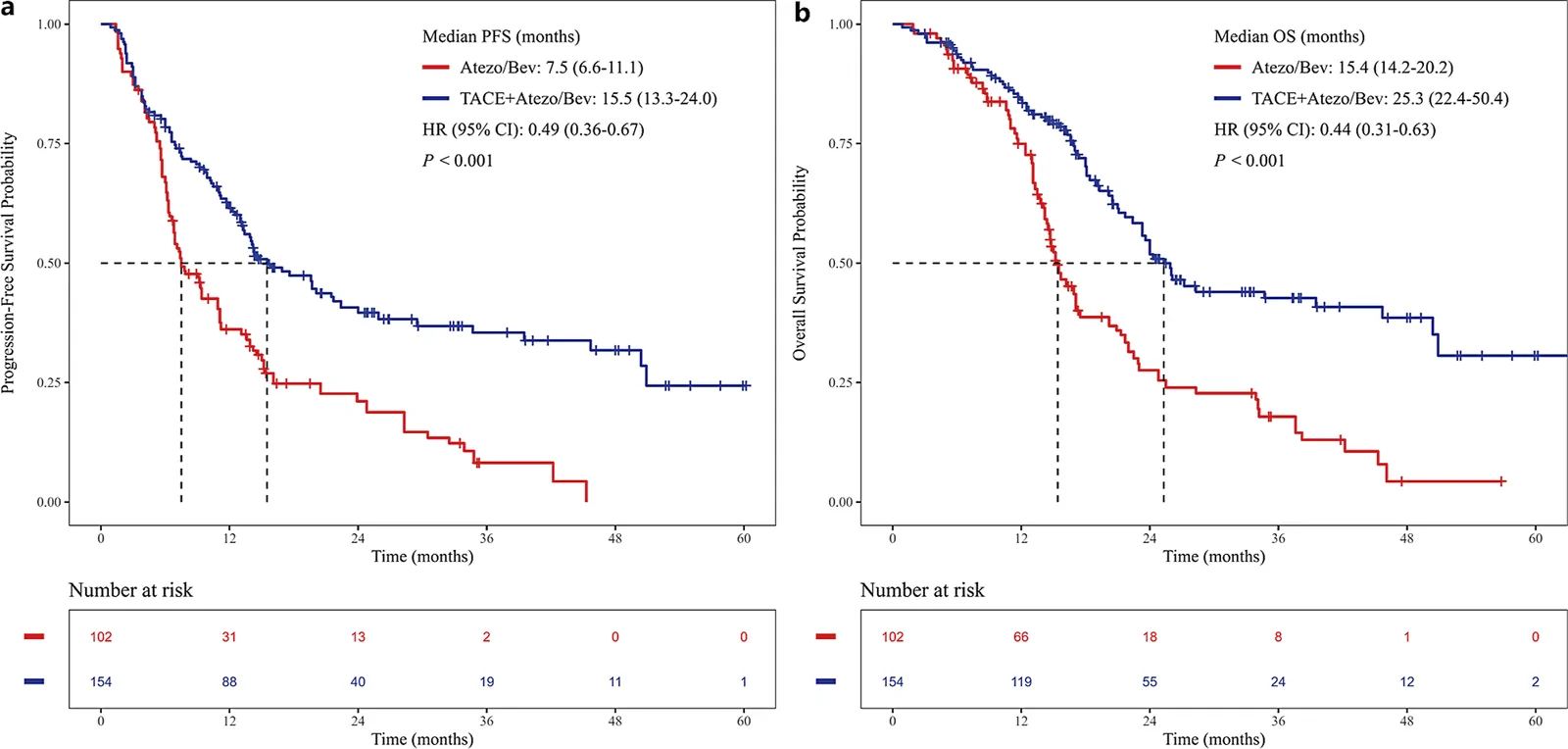
Kaplan-Meier curves of progression-free survival (**a**) and overall survival (**b**) in the two groups after stabilized inverse probability of treatment weighting. Atezo/Bev, atezolizumab and bevacizumab; TACE, transarterial chemoembolization.

Comparison between the cTACE and DEB-TACE subgroups within the TACE+Atezo/Bev group revealed no significant differences in median PFS (15.8 months [95% CI: 12.3–NR] vs. 14.3 months [95% CI: 11.9–25.9]; *p* = 0.572; HR: 1.13, 95% CI: 0.75–1.70) and OS (24.0 months [95% CI: 18.1–NR] vs. 26.1 months [95% CI: 21.7–NR]; *p* = 0.399; HR: 0.82, 95% CI: 0.51–1.30) (online suppl. Fig. S6). After multivariable adjustment, no significant differences in median PFS or OS were observed between the cTACE and DEB-TACE subgroups within the TACE plus Atezo/Bev group (online suppl. Fig. S7).

In the TACE+Atezo/Bev group, 50 patients (32.5%) received 1–2 TACE sessions and 104 (67.5%) received ≥ 3 sessions. No significant differences in median PFS or OS were observed for 1-2 versus ≥ 3 TACE sessions (PFS, 15.8 vs. 14.2 months, *p* = 0.200; OS, 24.3 vs. 25.3 months, *p* = 0.675) (online suppl. Fig. S8). TACE was performed before Atezo/Bev initiation in 139 patients (90.3%), with a median interval of 7.0 days, and after Atezo/Bev initiation in 15 patients (9.7%), with a median interval of 9.0 days. No significant differences in median PFS or OS were observed for TACE before versus after Atezo/Bev initiation (PFS, 14.3 vs. 34.7 months, *p* = 0.685; OS, 24.3 vs. 34.7 months, *p* = 0.605) (online suppl. Fig. S9).

### Analysis of Prognostic Factors

The prognostic factors identified by univariate and multivariable analyses for both PFS and OS are summarized in Table 2. The following factors were independently associated with prognosis on multivariable analysis: for PFS, ECOG PS (1 vs. 0) (HR = 1.64 [95% CI: 1.21–2.22]; *p* = 0.002), tumour size (≥5 vs. <5 cm) (HR = 2.37 [95% CI: 1.61–3.47]; *p* < 0.001), tumour number (>3 vs. ≤3) (HR = 1.63 [95% CI: 1.15–2.30]; *p* = 0.006), EHS (Present vs. Absent) (HR = 1.81 [95% CI: 1.32–2.49]; *p* < 0.001), AFP (≥400 vs. <400 ng/mL) (HR = 1.45 [95% CI: 1.07–1.98]; *p* = 0.017), and treatment (TACE+Atezo/Bev vs. Atezo/Bev) (HR = 0.45 [95% CI: 0.33–0.62]; *p* < 0.001) were independent prognostic factors; for OS, ECOG PS (1 vs. 0) (HR = 1.52 [95% CI: 1.09–2.14]; *p* = 0.015), tumour size (≥5 vs. <5 cm) (HR = 2.84 [95% CI: 1.83–4.39]; *p* < 0.001), tumour number (>3 vs. ≤3) (HR = 1.91 [95% CI: 1.27–2.87]; *p* = 0.002), EHS (Present vs. Absent) (HR = 1.72 [95% CI: 1.21–2.44]; *p* = 0.003), AFP (≥400 vs. <400 ng/mL) (HR = 1.50 [95% CI: 1.07–2.12]; *p* = 0.020), and treatment (TACE+Atezo/Bev vs. Atezo/Bev) (HR = 0.45 [95% CI: 0.32–0.63]; *p* < 0.001) were independent prognostic factors.

**Table 2.** Predictors of progression-free survival and overall survival in the primary cohort

Characteristics	Univariate analysis			Multivariate analysis		
	HR	95% CI	*p* value	HR	95% CI	*p* value
PFS analyses						
ECOG PS (1 vs. 0)	1.76	1.31–2.37	<0.001	1.64	1.21–2.22	0.002
Aetiology (HBV vs. others)	1.33	0.95–1.86	0.100	​	​	​
Cirrhosis (present vs. absent)	1.12	0.80–1.57	0.515	​	​	​
Tumour size (cm) (≥5 vs. <5)	1.91	1.34–2.73	<0.001	2.37	1.61–3.47	<0.001
Tumour number (>3 vs. ≤ 3)	1.58	1.12–2.23	0.010	1.63	1.15–2.30	0.006
PVTT (present vs. absent)	1.07	0.77–1.48	0.687	​	​	​
EHS (present vs. absent)	1.76	1.30–2.39	<0.001	1.81	1.32–2.49	<0.001
Child-Pugh class (B vs. A)	1.16	0.81–1.67	0.427	​	​	​
AFP (ng/mL) (≥400 vs. <400)	1.60	1.19–2.16	0.002	1.45	1.07–1.98	0.017
Treatment (TACE+Atezo/Bev vs. Atezo/Bev)	0.50	0.37–0.68	<0.001	0.45	0.33–0.62	<0.001
OS analyses						
ECOG PS (1 vs. 0)	1.73	1.25–2.41	0.001	1.52	1.09–2.14	0.015
Aetiology (HBV vs. others)	1.27	0.88–1.84	0.205	​	​	​
Cirrhosis (present vs. absent)	1.14	0.77–1.67	0.520	​	​	​
Tumour size (cm) (≥5 vs. <5)	2.36	1.58–3.55	<0.001	2.84	1.83–4.39	<0.001
Tumour number (>3 vs. ≤ 3)	1.93	1.29–2.89	0.001	1.91	1.27–2.87	0.002
PVTT (present vs. absent)	1.19	0.83–1.70	0.351	​	​	​
EHS (present vs. absent)	1.56	1.11–2.18	0.010	1.72	1.21–2.44	0.003
Child-Pugh class (B vs. A)	1.34	0.90–2.00	0.146	​	​	​
AFP (ng/mL) (≥400 vs. <400)	1.78	1.28–2.49	<0.001	1.50	1.07–2.12	0.020
Treatment (TACE+Atezo/Bev vs. Atezo/Bev)	0.46	0.33–0.65	<0.001	0.45	0.32–0.63	<0.001

HR, hazard ratio; CI, confidence interval; PFS, progression-free survival; ECOG PS, Eastern Cooperative Oncology Group performance status; HBV, hepatitis B virus; PVTT, portal vein tumoral thrombosis; EHS, extrahepatic spread; AFP, alpha-fetoprotein; TACE, transarterial chemoembolization; Atezo/Bev, atezolizumab plus bevacizumab; OS, overall survival.

The benefits of TACE+Atezo/Bev over Atezo/Bev in terms of both PFS and OS were consistent across most patient subgroups, with the notable exception of the subgroup with ascites (PFS, HR = 0.71 [95% CI: 0.44–1.17], *p* = 0.181; OS, HR = 0.62 [95% CI: 0.37–1.04], *p* = 0.072) (Fig. 4). In additional subgroup Kaplan-Meier analyses, TACE+Atezo/Bev also improved median PFS and OS in the PVTT subgroup (PFS, 14.3 vs. 7.5 months, HR = 0.48, *p* < 0.001; OS, 25.3 vs. 15.2 months, HR = 0.42, *p* < 0.001) and the EHS subgroup (PFS, 11.7 vs. 7.4 months, HR = 0.67, *p* = 0.037; OS, 21.7 vs. 15.2 months, HR = 0.52, *p* = 0.003) (online suppl. Figs. S10 and S11).

**Fig. 4.**
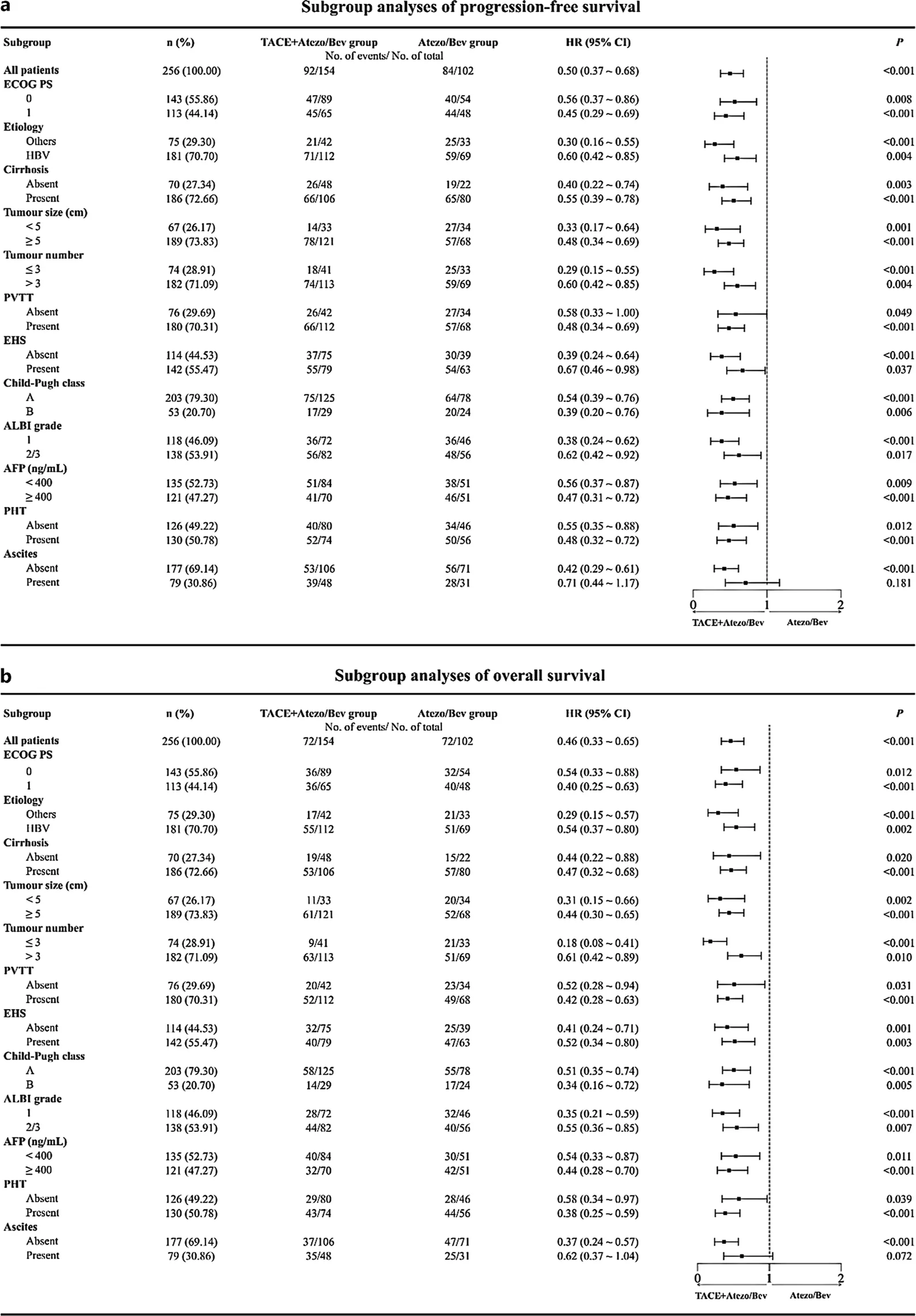
Subgroup analysis of progression-free survival (**a**) and overall survival (**b**). TACE, transarterial chemoembolization; Atezo/Bev, atezolizumab and bevacizumab; HR, hazard ratio; CI, confidence interval; ECOG PS, Eastern Cooperative Oncology Group performance status; PVTT, portal vein tumoural thrombosis; EHS, extrahepatic spread; ALBI, albumin-bilirubin; AFP, alpha-fetoprotein; PHT, portal hypertension.

### Safety

In the TACE+Atezo/Bev group, 119 (77.3%) patients experienced AEs of any grade, compared with 74 (72.5%) in the Atezo/Bev group (Table 3). Grade 3/4 AEs were observed in 60 (39.0%) patients in the TACE+Atezo/Bev group and 34 (33.3%) in the Atezo/Bev group. Increased blood bilirubin (9.1% vs. 7.8%, *p* = 0.727), alanine transaminase (ALT) (9.1% vs. 6.9%, *p* = 0.525) and aspartate aminotransferase (AST) (15.6% vs. 6.9%, *p* = 0.036) were the most common grade 3/4 AEs in the TACE+Atezo/Bev and Atezo/Bev groups. Among the 84 patients with Vp3/4 PVTT in the TACE+Atezo/Bev group, 17 patients (20.2%) experienced at least one of the following grade 3/4 AEs: increased blood bilirubin in 9 patients (10.7%), decreased albumin in 4 patients (4.8%), increased ALT in 8 patients (9.5%), and increased AST in 15 patients (17.9%). No mortality was attributed to the treatment. No significant differences were observed between the TACE+Atezo/Bev and Atezo/Bev groups in the incidence of any grade (13.6% vs. 12.7%, *p* = 0.837) or grade 3/4 (6.5% vs. 5.9%, *p* = 0.843) gastrointestinal haemorrhage. The incidence of TRAEs according to TACE type within the TACE+Atezo/Bev group is summarized in online supplementary Table 4, with no significant differences observed between the cTACE and DEB-TACE subgroups in any grade or grade 3/4 AEs.

**Table 3.** Treatment-related adverse events in the primary cohort

Events, *n* (%)	TACE+Atezo/Bev (*N* = 154)			Atezo/Bev (*N* = 102)			*p* value		
	any grade	Grade 1/2	Grade 3/4	any grade	Grade 1/2	Grade 3/4	any grade	Grade 1/2	Grade 3/4
Any TRAE	119 (77.3)	114 (74.0)	60 (39.0)	74 (72.5)	69 (67.6)	34 (33.3)	0.390	0.268	0.360
Fatigue	46 (29.9)	43 (27.9)	3 (1.9)	23 (22.5)	21 (20.6)	2 (2.0)	0.196	0.185	0.999
Decreased appetite	39 (25.3)	37 (24.0)	2 (1.3)	19 (18.6)	19 (18.6)	0 (0.0)	0.210	0.306	0.519
Abdominal pain	55 (35.7)	46 (29.9)	9 (5.8)	21 (20.6)	18 (17.6)	3 (2.9)	0.010	0.027	0.373
Nausea	19 (12.3)	18 (11.7)	1 (0.6)	13 (12.7)	12 (11.8)	1 (1.0)	0.923	0.985	0.999
Diarrhoea	37 (24.0)	35 (22.7)	2 (1.3)	19 (18.6)	19 (18.6)	0 (0.0)	0.306	0.431	0.519
Rash	13 (8.4)	13 (8.4)	0 (0.0)	8 (7.8)	8 (7.8)	0 (0.0)	0.864	0.864	–
Pruritus	20 (13.0)	19 (12.3)	1 (0.6)	11 (10.8)	10 (9.8)	1 (1.0)	0.597	0.531	0.999
Gastrointestinal haemorrhage	21 (13.6)	11 (7.1)	10 (6.5)	13 (12.7)	7 (6.9)	6 (5.9)	0.837	0.932	0.843
Epistaxis	7 (4.5)	7 (4.5)	0 (0.0)	5 (4.9)	5 (4.9)	0 (0.0)	0.999	0.999	–
Hypothyroidism	15 (9.7)	13 (8.4)	2 (1.3)	13 (12.7)	9 (8.8)	4 (3.9)	0.451	0.915	0.220
Fever	34 (22.1)	28 (18.2)	6 (3.9)	20 (19.6)	18 (17.6)	2 (2.0)	0.635	0.913	0.483
Hypertension	34 (22.1)	22 (14.3)	12 (7.8)	14 (13.7)	9 (8.8)	5 (4.9)	0.094	0.190	0.363
Proteinuria	18 (11.7)	12 (7.8)	6 (3.9)	10 (9.8)	7 (6.9)	3 (2.9)	0.636	0.781	0.999
Increased blood bilirubin	46 (29.9)	32 (20.8)	14 (9.1)	22 (21.6)	14 (13.7)	8 (7.8)	0.141	0.150	0.727
Decreased albumin	33 (21.4)	27 (17.5)	6 (3.9)	15 (14.7)	12 (11.8)	3 (2.9)	0.177	0.209	0.999
Increased ALT	45 (29.2)	31 (20.1)	14 (9.1)	18 (17.6)	11 (10.8)	7 (6.9)	0.035	0.048	0.525
Increased AST	57 (37.0)	33 (21.4)	24 (15.6)	19 (18.6)	12 (11.8)	7 (6.9)	0.002	0.047	0.036
Thrombocytopaenia	20 (13.0)	13 (8.4)	7 (4.5)	12 (11.8)	9 (8.8)	3 (2.9)	0.772	0.915	0.744
Immune pneumonia	2 (1.3)	2 (1.3)	0 (0.0)	2 (2.0)	1 (1.0)	1 (1.0)	0.652	0.999	0.398

For each adverse event category, grade 1/2 and grade 3/4 events were recorded and reported separately. TACE, transarterial chemoembolization; Atezo/Bev, atezolizumab plus bevacizumab; TRAE, treatment-related adverse event; ALT, alanine transaminase; AST, aspartate aminotransferase.

## Discussion

The findings of this nationwide multicenter retrospective cohort study indicated that the addition of TACE to Atezo/Bev significantly improved ORR (48.7% vs. 31.4%, *p* = 0.009), PFS (14.7 vs. 7.5 months; *p* < 0.001), and OS (24.3 vs. 15.2 months; *p* < 0.001) with an acceptable safety profile in patients with BCLC stage C HCC, compared with Atezo/Bev alone. Subgroup analysis revealed consistent survival benefits for the TACE+Atezo/Bev group across most clinical subgroups. The robustness of our findings was further supported by the use of both PSM and sIPTW to minimize potential confounding bias.

The Chance2201 study explored the efficacy difference of adding TACE to immune checkpoint inhibitors (ICIs) plus anti-VEGF antibody/tyrosine kinase inhibitors (TKIs) in the treatment of BCLC stage C HCC [23]. The median PFS and OS in the ICIs plus anti-VEGF antibody/TKIs group were 7.4 and 15.9 months, respectively, which were comparable to those in the Atezo/Bev group of our study. In patients with unresectable HCC enrolled in the IMbrave150 trial, the median PFS and OS with Atezo/Bev were 6.9 and 19.2 months, respectively [9]. In this study, the median PFS in the Atezo/Bev group was similar to that in the IMbrave 150 trial, while the median OS was shorter; this may be explained by the exclusive inclusion of BCLC stage C patients in our study. A multicenter retrospective study concluded that combining TACE with Atezo/Bev could improve the efficacy in unresectable HCC [24]. For patients with BCLC stage C HCC, subgroup analysis revealed that the median PFS and OS in the Atezo/Bev group were 6.5 and 14.3 months, respectively, which were slightly lower than those in our study. In addition, a multicenter retrospective study reported longer PFS and OS with on-demand TACE during Atezo/Bev therapy than with Atezo/Bev alone, although patients with BCLC stage C HCC accounted for only a minority of the cohort [25]. In this study, we further explored BCLC stage C HCC, and the results showed that TACE+Atezo/Bev significantly improved the median PFS and OS compared with Atezo/Bev alone.

The combination of TACE and systemic therapy has a plausible biological basis, but the potential synergistic effect should be interpreted cautiously. TACE can reduce intrahepatic tumour burden through chemoembolization and may induce tumour necrosis, with subsequent release of inflammatory mediators and tumour associated antigens that may contribute to immune activation [18, 26]. While TACE may contribute to tumour control by embolizing tumour feeding arteries, it can also induce an ischaemic hypoxic microenvironment that may upregulate VEGF expression [8, 14]. Elevated VEGF has been associated with an immunosuppressive tumour microenvironment through impaired dendritic cell differentiation and activation and recruitment of immunosuppressive cells, and has also been associated with shorter overall survival in HCC patients [27–30]. Bevacizumab may attenuate VEGF driven immunosuppression and promote vascular normalization, thereby facilitating immune cell infiltration and antitumour immune responses, which may in turn help maintain vascular normalization [7, 29]. Taken together, these findings provide biological plausibility for combining locoregional tumour control with systemic therapy, while the clinical relevance of this potential interaction requires further validation.

Two global phase III studies (LEAP-012 and EMERALD-1) have demonstrated the efficacy of TACE combined with targeted therapy and immunotherapy in unresectable HCC [31, 32]. Of note, the LEAP-012 study did not include patients with PVTT or EHS, and the EMERALD-1 study only enrolled patients with PVTT of VP1-2 grade [31, 32]. Nonetheless, the median PFS with TACE+Atezo/Bev in our study was 14.7 months, which was analogous to the reported median PFS of 14.6 and 15.0 months in LEAP-012 and EMERALD-1 studies, respectively. However, recently presented final OS results from EMERALD-1 showed no survival benefit with durvalumab plus bevacizumab plus TACE compared with TACE alone (median OS, 29.9 vs. 33.3 months; HR, 1.10; 95% CI, 0.87–1.39; *p* = 0.470), indicating that the previously observed PFS improvement did not translate into prolonged survival [33]. The EMERALD-3 trial demonstrated significantly longer PFS with tremelimumab plus durvalumab (STRIDE) combined with lenvatinib and TACE than with TACE alone (median PFS, 13.0 vs. 9.8 months; HR, 0.70; 95% CI, 0.57–0.86; *p* = 0.0007), whereas the immature OS analysis suggested a trend toward improved survival (median OS, 39.5 vs. 34.7 months; HR, 0.84; 95% CI, 0.65–1.09; *p* = 0.1814) [34]. Although not formally tested at this analysis, STRIDE combined with TACE showed a clinically meaningful improvement in PFS (HR, 0.71; 95% CI, 0.56–0.91) and a favourable trend in OS (HR, 0.70; 95% CI, 0.51–0.95) compared with TACE alone. The favourable findings from EMERALD-3 may broaden the range of promising systemic partners for TACE, with dual immune checkpoint blockade emerging as a potential strategy, although mature long-term data are needed. In addition, the recent TALENTACE study has reported that TACE combined with Atezo/Bev achieved the median PFS of 11.3 months in the treatment of HCC with moderate-to-high tumour burden (maximum tumour diameter + number of lesions ≥ 6), including unresectable BCLC stage A/B and BCLC stage C with ECOG PS 1 or VP1-2, which was lower than that in our study [35]. However, the CHANCE023 study demonstrated that TACE combined with Atezo/Bev achieved the median PFS of 19.23 months and the median OS of 29.97 months in patients with unresectable HCC, while both the median PFS and OS in CHANCE023 were longer than those in our study, which may be due to the fact that CHANCE023 is a retrospective study and included more non-BCLC stage C patients than our study [36]. In patients with BCLC stage C HCC, a multicenter retrospective subgroup analysis reported that TACE+Atezo/Bev yielded median PFS of 12.8 months and median OS of 25.2 months, representing a shorter PFS but comparable OS relative to our findings [24].

This study investigated the impact of TACE combined with Atezo/Bev on survival benefits across various clinical subgroups. However, the subgroup analysis results were exploratory and required cautious interpretation. TACE serves as the standard treatment for BCLC stage B HCC, while it is also extensively applied in BCLC stage C to decrease intrahepatic tumour burden [6, 37]. With the alleviation of tumour burden, the efficacy of Atezo/Bev treatment may be further improved, and this phenomenon may be observed in patients stratified by tumour size (≥5 cm), tumour number (>3), and AFP (≥400 ng/mL) subgroups. In this study, over half of the patients were complicated with PVTT, which may benefit from TACE as it is supplied by arterial blood [38]. In addition to liver cirrhosis, PHT is also associated with PVTT formation; therefore, TACE combined with Atezo/Bev may improve the efficacy in patients with PHT. Patients with Child-Pugh class A or B liver function have been proven to have comparable tolerance to Atezo/Bev treatment, but a major concern associated with TACE combined with Atezo/Bev is the possible worsening of liver function, especially in patients with Child-Pugh class B liver function [39]. In this study, TACE combined with Atezo/Bev was associated with improved survival outcomes, with no obvious additional risks observed. These findings suggested that TACE combined with Atezo/Bev might be a more effective treatment for BCLC stage C HCC, but further prospective multicenter randomized clinical trials are still needed to confirm this.

Regarding safety, there was no significant difference in the incidence of any grade (77.3% vs. 72.5%, *p* = 0.390) and grade 3/4 (39.0% vs. 33.3%, *p* = 0.360) AEs between the TACE+Atezo/Bev group and Atezo/Bev group in this study, with the majority of AEs being readily manageable. In the TALENTACE study, the incidence of grade 3–5 AEs in the TACE+Atezo/Bev group was 63.8%, which was significantly higher than that in this study (39.0%), and this discrepancy may be attributed to the retrospective nature of this study [35]. Compared with the Atezo/Bev group, patients in the TACE+Atezo/Bev group had higher incidence of any grade abdominal pain (35.7% vs. 20.6%, *p* = 0.010), increased ALT (29.2% vs. 17.6%, *p* = 0.035), and increased AST (37.0% vs. 18.6%, *p* = 0.002), as well as a higher incidence of grade 3/4 increased AST (15.6% vs. 6.9%, *p* = 0.036), which may be related to TACE-associated liver injury [40]. Notably, no significant differences existed between the TACE+Atezo/Bev and Atezo/Bev groups in terms of the incidence of any grade (13.6% vs. 12.7%, *p* = 0.837) or grade 3/4 gastrointestinal haemorrhage (6.5% vs. 5.9%, *p* = 0.843), which is consistent with the conclusions of previous study [24]. In general, combination TACE and Atezo/Bev was well tolerated in patients with BCLC stage C HCC.

This study was subject to certain limitations. First, although PSM and sIPTW were applied to improve baseline comparability between groups, unmeasured confounding could not be completely excluded. Because the indication for TACE was determined by real-world clinical considerations, residual indication bias could not be completely excluded despite the additional propensity score and sensitivity analyses. The analyses according to TACE session number and timing were exploratory and required cautious interpretation because of potential survivor bias and the limited number of patients who received TACE after Atezo/Bev initiation. Second, this study was conducted across multiple centres nationwide. Despite training provided to data collectors before data collection, inconsistencies might still exist. Additionally, it is possible that the concurrent COVID-19 pandemic limited the comprehensiveness of patient assessments, even with a meticulous review of electronic medical records. Furthermore, although TACE procedures were performed in accordance with guidelines at each centre, heterogeneity in TACE procedures might still exist among different centres. The comparison of cTACE and DEB-TACE was exploratory, and the available sample size was insufficient to draw definitive conclusions regarding differences between the two embolization techniques. Third, given the exclusively Chinese cohort and its hepatitis B virus (HBV)-predominant aetiology, this conclusion warrants validation in other geographic and etiological populations. In conclusion, for patients with BCLC stage C HCC, TACE may further enhance the efficacy of Atezo/Bev with an acceptable safety profile, making the “TACE+Atezo/Bev” regimen a promising treatment option for them.

## Acknowledgement

We thank all the investigators at each site, as well as the staff involved in this study.

## Statement of Ethics

This retrospective study was conducted in accordance with the Declaration of Helsinki and approved by the Ethics Committee of the National Cancer Center/Cancer Hospital, Chinese Academy of Medical Sciences and Peking Union Medical College (ethical review Nos. 25/241-0241 and 25/654-5600), which waived the requirement for written informed consent. This study followed the Strengthening the Reporting of Observational Studies in Epidemiology (STROBE) statement, with the completed STROBE checklist provided as online supplementary material (for all online suppl. material, see https://doi.org/10.1159/lic/admag010).

## Conflict of Interest Statement

The authors declare no conflicts of interest.

## Funding Sources

This study was supported by CAMS Innovation Fund for Medical Sciences (CIFMS): 2024-I2M-C&T-B-073 and the National Natural Science Foundation of China, No. 82330061. The funders had no role in the study design, execution, or analysis; manuscript conception, planning, or writing; or the decision to publish.

## Author Contributions

X.L., X.-W.Z., Y.-F.X., X.-G.Y., C.L., R.-B.L., H.-B.S., T.-G.S., D.-P.F., J.W., Y.Z., Q.-D.L., H.-T.H., W.-J.F., and P.S. designed the study. Y.-G.L., L.-W.D., X.C., L.-N.Y., Y.W., Y.C., T.-S.L., Y.X., T.-Y.H., J.X., R.-C.N., L.-J.S., and Z.-M.X. collected the data. X.-W.Z., L.-W.D. and Y.-G.L. analysed the data, wrote the original draft, and have accessed and verified the data. X.L. and X.-W.Z. provided supervision. All authors reviewed and approved the final version of the manuscript.

## Data Availability

The data that support the findings of this study are not publicly available due to the Personal Information Protection Act but are available from the corresponding author upon reasonable request.
